# Hormonal profiles of men with highly elevated SHBG and primary testicular dysfunction support free hormone hypothesis

**DOI:** 10.1210/jendso/bvag185

**Published:** 2026-08-12

**Authors:** Thiago Gagliano-Jucá, Ishika Puri, Gabriel Ramirez-Rivera, Skand Shekhar, Rodrigo J Valderrábano, Anna L Goldman, Yusnie Memish-Beleva, Mary Weiss, Yili Valentine Shang, Karol M Pencina, Ravi Jasuja, Shalender Bhasin

**Affiliations:** Research Program in Men's Health: Aging and Metabolism, Brigham and Women's Hospital, Harvard Medical School, Boston, MA 02115, USA; Research Program in Men's Health: Aging and Metabolism, Brigham and Women's Hospital, Harvard Medical School, Boston, MA 02115, USA; Research Program in Men's Health: Aging and Metabolism, Brigham and Women's Hospital, Harvard Medical School, Boston, MA 02115, USA; Research Program in Men's Health: Aging and Metabolism, Brigham and Women's Hospital, Harvard Medical School, Boston, MA 02115, USA; Research Program in Men's Health: Aging and Metabolism, Brigham and Women's Hospital, Harvard Medical School, Boston, MA 02115, USA; Research Program in Men's Health: Aging and Metabolism, Brigham and Women's Hospital, Harvard Medical School, Boston, MA 02115, USA; Research Program in Men's Health: Aging and Metabolism, Brigham and Women's Hospital, Harvard Medical School, Boston, MA 02115, USA; Research Program in Men's Health: Aging and Metabolism, Brigham and Women's Hospital, Harvard Medical School, Boston, MA 02115, USA; Research Program in Men's Health: Aging and Metabolism, Brigham and Women's Hospital, Harvard Medical School, Boston, MA 02115, USA; Research Program in Men's Health: Aging and Metabolism, Brigham and Women's Hospital, Harvard Medical School, Boston, MA 02115, USA; Research Program in Men's Health: Aging and Metabolism, Brigham and Women's Hospital, Harvard Medical School, Boston, MA 02115, USA; Research Program in Men's Health: Aging and Metabolism, Brigham and Women's Hospital, Harvard Medical School, Boston, MA 02115, USA

**Keywords:** high sex hormone-binding globulin levels, free hormone hypothesis, free testosterone, biologically active fraction of hormones, diagnosis of hypogonadism, free testosterone measurement

## Abstract

**Context:**

Free hormone hypothesis (FHH) posits that free testosterone crosses plasma membrane to activate intracellular signaling and is biologically active fraction of circulating testosterone. Widely accepted in other fields, FHH has been debated in andrology.

**Objective:**

To disentangle the roles of free and total testosterone, we studied men with greatly elevated sex hormone-binding globulin (SHBG) levels and primary hypogonadism, who had marked divergence in total and free testosterone concentrations, in whom elevated gonadotropins provided independent evidence of primary testicular insufficiency.

**Design:**

Prospective hormonal evaluation of men with greatly elevated SHBG.

**Outcomes:**

Total and free testosterone, LH, FSH, symptom ascertainment

**Results:**

Among 20 men with SHBG>100 nmol/L, 15 met Endocrine Society's criteria for primary hypogonadism: low free testosterone, one or more symptoms/signs, elevated LH and/or FSH. Among 15 men with elevated LH and/or FSH and unequivocally low free testosterone who met the criteria for primary hypogonadism, 4 had total testosterone that exceeded the reference range (>916 ng/dL) and 8 had total testosterone >450 ng/dL, substantially above the lower normal limit.

**Conclusion:**

In men with markedly elevated SHBG, in whom total and free testosterone diverged substantially, low free testosterone was associated with elevated gonadotropins, providing independent evidence of primary testicular insufficiency even when total testosterone levels were above the reference range or substantially above the lower normal limit. Although sample size was small, these illustrative “n-of-1” observations in uncommon patients are consistent with the primacy of free testosterone over total testosterone in the hypothalamic–pituitary–testicular feedback loop to regulate LH and FSH, and support the FHH.

Testosterone deficiency manifests as a clinical syndrome in men due to dysfunction at any level of the hypothalamic–pituitary–testicular (HPT) axis, leading to inadequate testicular secretion of testosterone (1). Therefore, accurate measurement of testosterone concentrations is essential for the diagnosis of testosterone deficiency. Circulating testosterone exists predominantly bound to sex hormone-binding globulin (SHBG) and albumin, with lesser fractions bound to orosomucoid and corticosteroid-binding globulin, and only 1-4% circulating as unbound free testosterone (2). The free hormone hypothesis (FHH) posits that the free testosterone is able to rapidly diffuse across the plasma membrane to activate intracellular signaling, producing tissue-specific effects, and is, therefore, the biologically active fraction of circulating testosterone (3, 4).

An important corollary of the FHH is that free rather than total testosterone serves as the key mediator, directly as well as through its conversion to estradiol, of the negative feedback on the HPT-axis through central biosensors in hypothalamic KNDy neurons and pituitary gonadotrophs. Consequently, when free testosterone concentration is within the physiological range—irrespective of SHBG or total testosterone concentrations—men should exhibit clinical eugonadism with normal luteinizing hormone (LH) and follicle-stimulating hormone (FSH) concentrations; conversely, subnormal free testosterone should drive compensatory elevations in LH and/or FSH, independent of total testosterone or SHBG.

An additional corollary of the FHH is that free testosterone concentration should outperform total testosterone concentration as a circulating biomarker of systemic androgen action. However, total and free testosterone concentrations display strong collinearity (r = 0.7-0.96 in healthy young men across assays), while associations between testosterone changes and testosterone-responsive endpoints such as sexual desire and sexual activity remain modest (r = 0.1-0.3) (5-8). For example, in the Testosterone Trials (TTrials) (6, 7), the increments in free testosterone concentrations above baseline correlated modestly more robustly with sexual activity improvements than changes in total testosterone, though both showed positive, SHBG-independent association—highlighting how collinearity between total and free testosterone confounds these correlational analyses in observational and interventional studies and fuels debates over the superiority of free testosterone over total testosterone (9).

To disentangle the relative roles of free testosterone vs total testosterone, we leveraged men with markedly elevated SHBG, who had marked discordance in total and free testosterone concentrations, as a natural experiment for FHH validation. Those with primary testicular failure and elevated SHBG proved especially illuminating, as their LH/FSH concentrations offer an endogenous readout of androgen bioactivity independent of exogenous confounders. Per FHH predictions, in men with intact HPT-axis with high SHBG, free testosterone would transiently decrease, leading to transient elevation of LH and FSH secretion and restoration of free testosterone concentrations into the normal range, yielding a steady-state characterized by high-normal or elevated total testosterone, normal free testosterone, and normal gonadotropin concentrations. In men with primary hypogonadism and high SHBG, FHH predicts: (1) low free testosterone; (2) variably elevated LH/FSH in proportion to the testicular impairment; and (3) total testosterone spanning low, normal to supraphysiologic levels, depending on the SHBG elevation and residual testicular function. We assumed *a priori* that the hormone levels from men with very high SHBG and central hypogonadism or men with very high SHBG and normal free testosterone levels will not be useful in this analysis because in such men, serum LH and FSH levels could not serve as sentinels of circulating androgen action.

The present manuscript presents data from a cohort of men with markedly elevated SHBG levels and wide divergence in total and free testosterone concentrations who were studied prospectively, using a prespecified structured protocol, approved by the institutional review board, to evaluate whether their clinical and hormonal findings conform to the predictions of the FHH. We did not include women in these analyses because it is not clear whether the circulating testosterone levels are regulated by similar feedback and feed-forward mechanisms within the hypothalamic–pituitary–ovarian axis as they are in men.

## Methods

The study protocol was approved by the Mass General Brigham (MGB) Human Subjects Protection Program. All participants provided written, informed consent.

### The participant selection and recruitment

We identified potential cases using the MGB Healthcare System Research Patient Data Registry (RPDR). The RPDR gathers clinical data from patients who have received clinical care at one of the several academic medical centers associated with the MGB healthcare system, and can identify patients with specific demographics, diagnoses, laboratory tests, medications, and procedures. With the goal of identifying men with greatly elevated SHBG and primary hypogonadism, we used the RPDR query tool to query for male patients seen between January 1, 2013 and February 5, 2023 who were aged 18 to 84 years, and had SHBG levels >100 nmol/L (reference range 13 to 71 nmol/L) in whom testosterone and other hormone levels were available. The resulting dataset underwent a rigorous manual chart review to ensure data accuracy and adherence to eligibility criteria.

**Figure 1:**
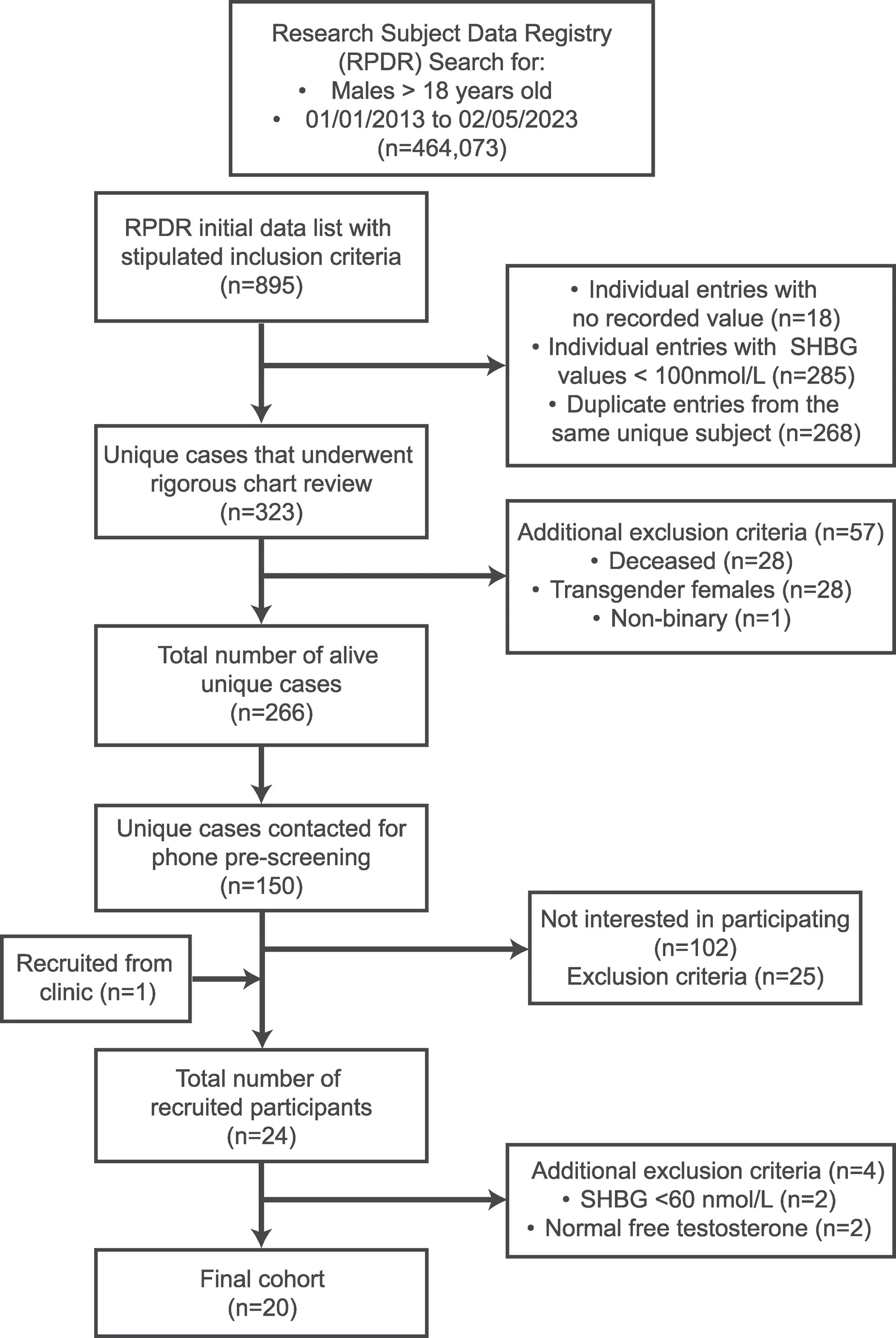
STROBE diagram showing the selection of the analytic sample.

Exclusion criteria included recent major illnesses or hospitalizations, active testosterone therapy or therapies known to affect androgen metabolism or SHBG levels. Among 895 individual male patients identified in the RPDR search, we excluded those with missing SHBG data (n = 18), SHBG values below the threshold (n = 285), duplicate entries (n = 268), deceased subjects (n = 28), and patients identified as transgender (n = 28) or gender diverse (n = 1), or other exclusionary illnesses. After these exclusions, 266 unique cases met the eligibility criteria and were eligible for telephone prescreening if their current contact information was available (Fig. 1). Of the 150 patients who were called, 102 declined participation or could not be contacted, 25 were deemed ineligible after telephone screening. One additional participant was recruited directly from the BWH Men's Health Clinic. A total of 24 participants, who met the eligibility criteria were invited for an in-person visit to our clinical research unit for detailed investigation.

**Figure 2:**
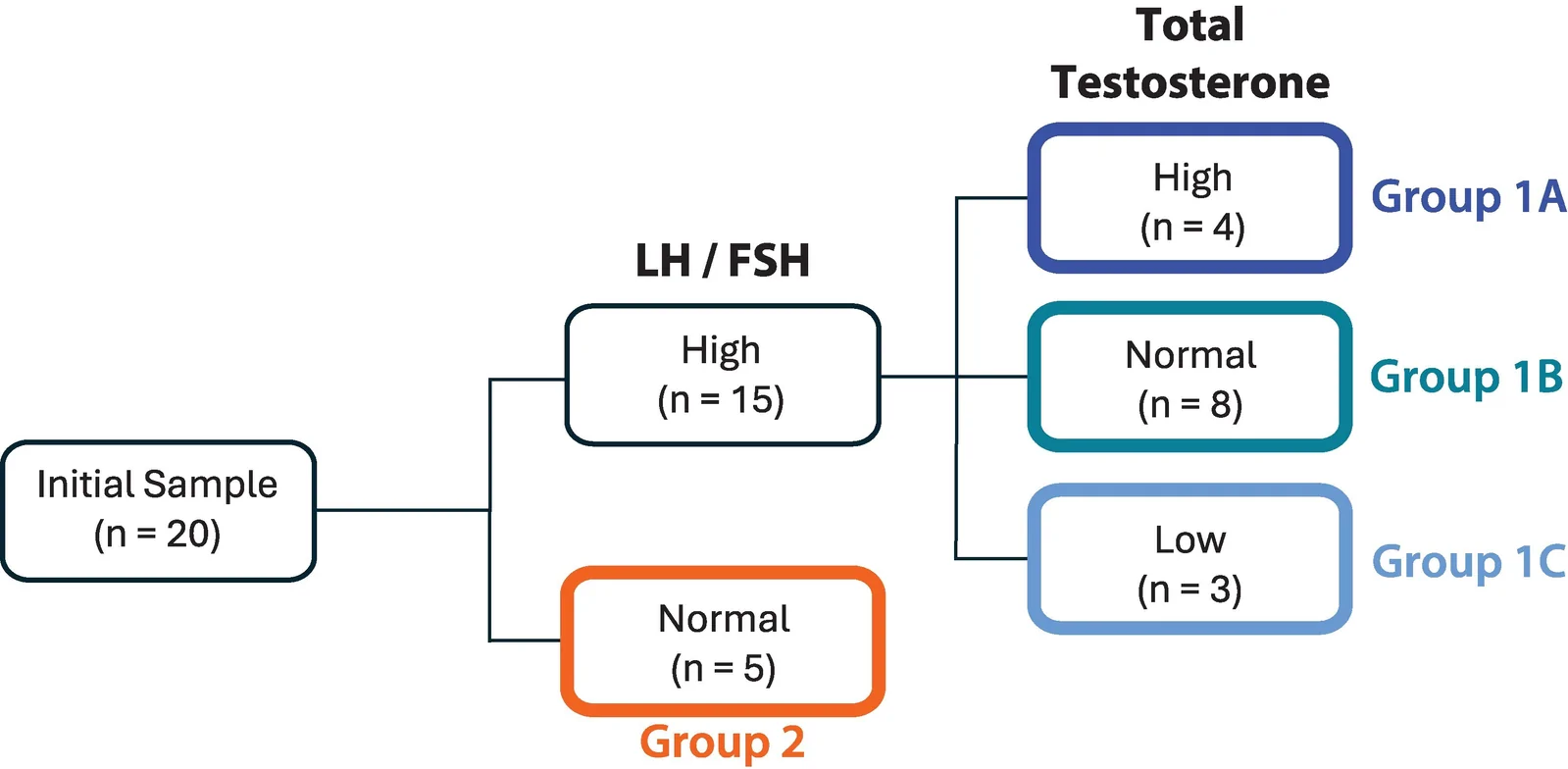
Classification of participants according to their gonadal status. Reference ranges: total testosterone, 264-916 ng/dL; free testosterone 66-309 pg/mL; LH, 1.7-8.6 IU/L; FSH 1.3-8.4 IU/L. Reference ranges are derived from healthy men are not age-adjusted (10).

The participants then had SHBG and other hormone levels measured in the Brigham Research Assay Core Laboratory. Two participants who were found to have SHBG <60 nmol/L were excluded; two additional participants who were found to have normal free testosterone were also excluded, leading to a final analytic sample of 20 men (Fig. 2).

### Data collection

The data collection during the in-person interview involved a comprehensive assessment following written informed consent. A structured medical history including medication history was obtained, and blood chemistries and hormone levels including TSH, free T4, IGF-1, and sex hormone levels were evaluated prospectively for known causes of elevated SHBG. The participants underwent a physical examination that included measurement of testicular volume using a Prader orchidometer and evaluation of gynecomastia. Fasting, morning venous blood samples were collected for biochemical evaluation.

### Laboratory evaluation

Serum total testosterone concentration was measured using a liquid chromatography tandem mass spectrometry (LC-MS/MS) assay, certified by the Centers for Disease Control and Prevention's (CDC) hormone standardization program for testosterone (HoST) (6, 11); the assay methods have been published (6, 11, 12). The lower limit of quantitation for total testosterone was 1 ng/dL, and the assay exhibited a linear range from 1 to 1000 ng/dL. In aliquots of charcoal stripped serum spiked with varying concentrations of testosterone, the recovery was 102 ± 2%. The laboratory is certified by the HoST Program of the CDC, an accuracy-based benchmark. The overall mean (95% confidence interval) bias from the HoST quality control pools was 0.7% (−2.3 to +3.6%) in the low range (−7.7 to 24.0 ng/dL); −0.8% (−2.7 to +1.0%) in the medium range (24.7 to 318.0 ng/dL); and 0.1% (−1.5 to +1.8%) in the high range (329 to 942 ng/dL). The reference range (264 to 916 ng/dL) for total testosterone assay using the HoST-certified LC-MS/MS assay has been published (11).

Free testosterone concentrations were measured using a standardized equilibrium dialysis method coupled with the measurement of testosterone in the dialysate using an LC-MS/MS assay, described above (10). The lower limit of the normal range for free testosterone in healthy nonobese men, 19 years or older, measured using this standardized equilibrium dialysis method, is 66 pg/mL (10). The age-stratified lower limit for healthy young men, 19 to <40 years of age was 119.6 pg/mL and in older men, 60 years or older (66.2 pg/mL) (10). The interassay CVs of the equilibrium dialysis assay were 11.5% and 14.5% in the two male quality control pools, 14.2% and 14.2% in the two female quality control pools, and 9.8% at the lower limit of quantitation (1 pg/mL); intra-assay CVs of the equilibrium dialysis assay were <10% (10).

Serum SHBG, LH, and FSH were measured by two-site directed immunochemiluminescent assays (Beckman Coulter, Brea, CA) (10, 12) (RRIDs for SHBG assay: AB_2893035; for LH: AB_2750984; and for FSH: AB_2750983). The lower limit of quantitation for the SHBG, LH, and FSH assays were 2.5 nmol/L, 0.1 U/L, and 0.1 U/L, respectively; the interassay CV for each of these hormones was <10%.

### Assessment of symptoms of testosterone deficiency

Questionnaires were used to objectively evaluate symptoms of testosterone deficiency. Erectile function was assessed using the erectile function domain of the International Index of Erectile Function (IIEF-EF) (score range 1 to 30); higher score indicates better erectile function, scores <25 correspond to erectile dysfunction (13). Sexual desire was assessed using the sexual desire domain of the DeRogatis interview for sexual function II (DISF-M-II) (score range 1 to 33); higher scores indicate greater sexual desire and scores ≤20 correspond to low libido (14). Fatigue was assessed using the functional assessment of chronic illness therapy (FACIT), fatigue subscale (score range 0 to 52; higher scores indicate less fatigue; scores ≤34 correspond to fatigue and scores of 30 or below indicate moderate to severe fatigue) (15). The depressive symptoms were assessed by the patient health questionnaire-9 (PHQ-9); scores range from 0 to 27 (0-4: minimal to no depressive symptoms, 5 to 9: mild, 10 to 14: moderate, 15 to 19: moderately severe, and 20 to 27: severe depressive symptoms) (16).

### Statistical analysis

The characteristics of study participants were summarized as means and standard deviations (SD). Graphs were generated using GraphPad Prism version 10.6.1. The association between serum SHBG and total testosterone concentrations within Group 1 was assessed by linear regression.

## Results

As described in the Methods section, among the 266 unique patients in the RPDR who underwent telephone prescreening (SROBE diagram, Fig. 1), 24 participants, who met the eligibility criteria, were invited for an in-person visit to our clinical research unit for detailed investigation. During this in-person evaluation, two participants, who were found to have SHBG <60 nmol/L in our research laboratory, were excluded (Fig. 1). An additional two participants had normal total and free testosterone concentrations, were deemed not to have hypogonadism, and were not considered for further analyses (Fig. 1).

**Figure 3:**
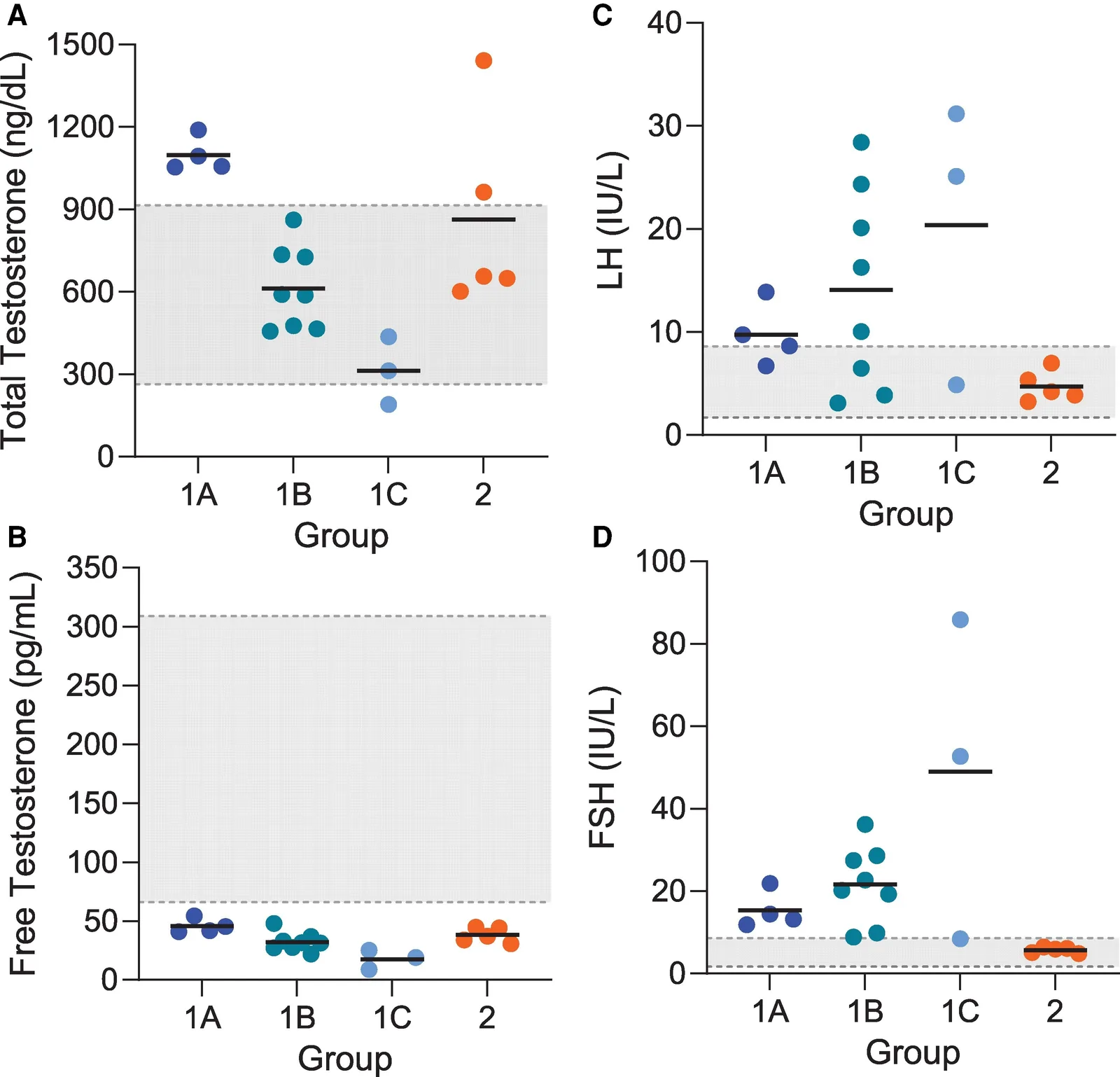
Hormone concentrations in each group. The shaded areas represent the normal range for each hormone; these reference ranges are not age-adjusted. Reference ranges: total testosterone, 264-916 ng/dL; free testosterone 66-309 pg/mL; LH, 1.7-8.6 IU/L; FSH 1.3-8.4 IU/L. FSH, follicle-stimulating hormone; LH, luteinizing hormone. (A) Total testosterone; (B) free testosterone; (C) LH; and (D) FSH.

The 20 participants with low free testosterone concentration were categorized by serum total testosterone, LH, and FSH levels to evaluate whether they had normal, low, or high levels of each of these hormones. We were particularly interested in men who had low serum free testosterone levels (<66 pg/mL) and elevated serum LH (normal range 1.7 to 8.6 U/L) and/or elevated FSH (normal range 1.3 to 8.4 U/L) because their elevated LH and/or FSH concentrations could provide independent evidence of primary testicular insufficiency. Among the 20 participants with elevated SHBG level and low free testosterone concentration, 15 participants had elevated serum LH and/or FSH concentrations (Group 1, Table 1), and five participants had LH and FSH concentrations within the normal male range (Group 2; Figs. 2 and 3). Four participants had history of cirrhosis, one had autoimmune hepatitis, one metabolic syndrome-associated liver disease, and one participant was taking phenytoin; the cause of elevated SHBG was not identified in the remaining 17 participants.

**Table 1:** Clinical characteristics of study participants

	Group 1A	Group 1B	Group 1C	Aggregate Group 1	Group 2
	N = 4	N = 8	N = 3	N = 15	N = 5
Age (years)	65 ± 7	71 ± 8	61 ± 15	68 ± 10	59 ± 11
Height (cm)	175.6 ± 2.7	174.9 ± 3.6	177.3 ± 6.3	176 ± 4	171.0 ± 5.5
Weight (kg)	76.3 ± 11.7	78.7 ± 12.0	72.6 ± 28.4	76.8 ± 14.9	103.4 ± 25.1
BMI (kg/m^2^)	24.7 ± 3.3	25.7 ± 3.8	23.1 ± 9.1	24.9 ± 4.7	35.7 ± 10.3
SHBG (nmol/L)	133.0 ± 20.2	98.4 ± 19.9	86.7 ± 11.0	105.3 ± 25.0	119.0 ± 48.9
Albumin (g/dL)	4.2 ± 0.5	4.3 ± 0.2	4.2 ± 0.8	4.2 ± 0.4	4.3 ± 0.4
Total testosterone (ng/dL)	1098.5 ± 63.0	612.8 ± 149.3	313.3 ± 123.5	682 ± 309	863 ± 354
Free Testosterone (pg/mL)	45.7 ± 6.2	32.2 ± 7.8	17.7 ± 8.2	33 ± 12	38 ± 6
FSH (IU/L)	15.3 ± 4.5	21.6 ± 9.3	49.0 ± 38.8	25 ± 20	5.7 ± 0.7
LH (IU/L)	9.7 ± 3.0	14.1 ± 9.6	20.4 ± 13.8	14 ± 9	4.7 ± 1.5
Hemoglobin (g/dL)	15.3 ± 1.7 (n = 2)	13.9 ± 0.9 (n = 7)	11.9 ± 0.6 (n = 2)	14 ± 1 (n = 11)	14.6 ± 0.8
Hematocrit (%)	45.5 ± 5.3 (n = 2)	42.3 ± 2.4 (n = 7)	35.9 ± 1.1 (n = 2)	42 ± 4 (n = 11)	44.1 ± 2.0
Fatigue (FACIT)	11.3 ± 3.3	12.9 ± 8.7	21.0 ± 5.3	14.1 ± 7.6	12.8 ± 4.7
Depression (PHQ-9)	2.8 ± 4.2	3.3 ± 4.2	7.0 ± 4.4	3.9 ± 4.3	3.2 ± 4.1
Erectile function (IIEF-EF)	20.0 ± 6.7	13.8 ± 12.3	19.0 ± 10.5	16.5 ± 10.5	18.0 ± 10.1
Sexual desire (DISF-SD)	24.8 ± 6.1	16.8 ± 5.7	20.0 ± 8.5	19.5 ± 6.9	23.8 ± 7.3

Data displayed as means ± SDs. FACIT, Functional Assessment of Chronic Illness Therapy, fatigue subscale (score range 0 to 52; higher scores indicate less fatigue); PHQ-9, patient health questionnaire-9 (scores range from 0 to 27, higher scores indicate worse depression); IIEF-EF, International Index of Erectile Function (score range 1 to 30; higher score indicates better erectile function), DISF-SD, DeRogatis Interview for Sexual Function II, sexual desire domain (score range 1 to 33; higher scores indicate greater sexual desire).

**Figure 4:**
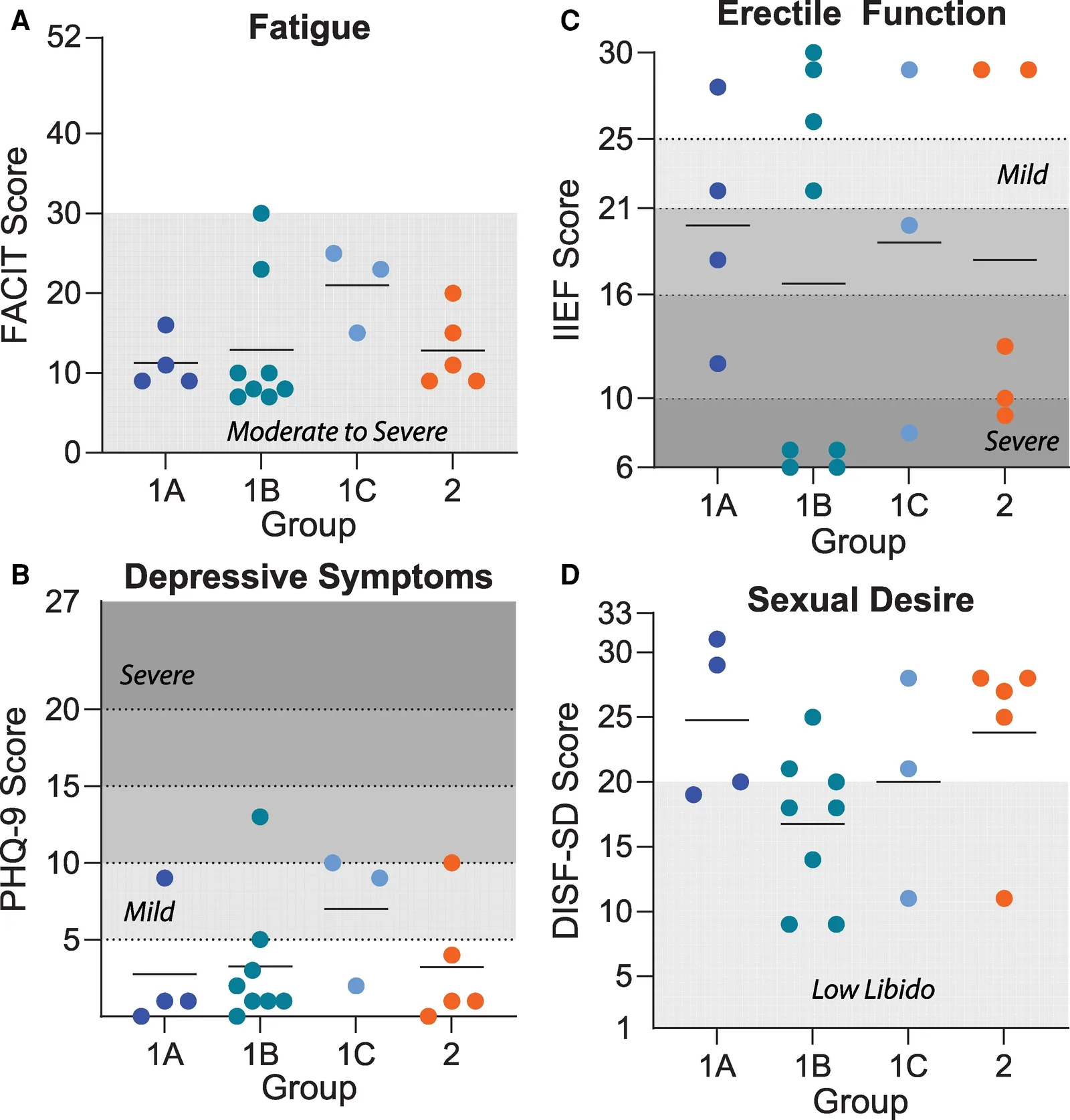
Questionnaire scores in each group. (A) FACIT, functional assessment of chronic illness therapy, fatigue subscale (score range 0 to 52; higher scores indicate less fatigue); (B) PHQ-9, patient health questionnaire-9 (scores range from 0 to 27, higher scores indicate worse depression); (C) IIEF-EF, International Index of Erectile Function (score range 1 to 30; higher score indicates better erectile function), (D) DISF-SD, DeRogatis Interview for Sexual Function II, sexual desire domain (score range 1 to 33; higher scores indicate greater sexual desire). The shaded areas in the top 4 panels represent scores that are out of the normal range (for FACIT-Fatigue score <30; PHQ-9 score >4; IIEF score <25; DISF sexual desire domain score <20).

The patients in group 1 (n = 15) had a mean ± SD age of 68 ± 10 years, body weight 76.8 ± 14.9 kg; body mass index 24.9 ± 4.7; SHBG 105.3 ± 25.0 nmol/L; serum total testosterone 682 ± 309 ng/dL; free testosterone 32.9 ± 12 pg/mL; FSH 25 ± 20 U/L; LH 14 ± 9 U/L; estradiol 33.9 ± 16.7 pg/mL; and dihydrotestosterone 70.5 ± 35.2 ng/dL (Table 2). All 15 patients in group 1 met the Endocrine Society's criteria for primary hypogonadism (1) as they had one or more symptoms (low libido, erectile dysfunction, fatigue, and/or depressive symptoms) verified by validated questionnaires (Fig. 4), low free testosterone concentration, and elevated LH and/or elevated FSH levels. Four of these 15 patients in group 1 are notable because their total testosterone concentrations were above the upper limit of the normal range for healthy young men (>916 ng/dL) and yet, they met the criteria for primary hypogonadism (1): one or more symptoms of hypogonadism, low free testosterone concentration, and elevated LH and/or FSH levels (Table 1). Three of these four patients had gynecomastia, and each had one or more symptoms of hypogonadism (Table 2). All had moderate to severe fatigue and low libido, and three had erectile dysfunction.

**Table 2:** Clinical characteristics of 15 individual participants in Group 1

			Group 1A				Group 1B								Group 1C		
	Subject ID		004	005	025	026	02	16	19	23	24	27	29	33	01	03	07
Demographics	Age (y)		73	57	65	66	69	74	55	67	70	81	76	78	72	44	68
	Height (cm)		176	175	179	172.5	169	175.5	176.5	175.5	178.5	170	179	175.5	177.5	183.5	171
	Weight (kg)		69.9	84.2	87.8	63.1	70.2	81.9	61.6	103.1	77.9	73.4	81.5	79.9	105.3	53.7	58.8
	BMI (kg/m^2^)		22.6	27.5	27.4	21.2	24.6	26.6	19.8	33.5	24.4	25.4	25.4	25.9	33.4	15.9	20.1
Physical exam	Gynecomastia		Yes	Yes	No	Yes	Yes	No	Yes	Yes	Yes	Yes	No	No	No	No	No
	Left Testis (mL)		25	18	12	20	20	20	—	22	20	12	20	25	12	10	5
	Right Testis (mL)		25	18	12	—	20	20	15	14	20	12	20	25	15	10	5
Labs	SHBG (nmol/L)		142	140	147	103	119	89	105	87	81	117	68	121	98	86	76
	Total Testosterone (ng/dL)		1189	1094	1054	1057	736	588	477	465	457	727	590	862	190	437	313
	Free Testosterone (pg/mL)		45.4	41.0	41.9	54.5	31.3	33.0	22.1	27.6	27.2	31.7	48.0	36.8	8.9	25.1	19.2
	FSH (IU/L)		14.4	13.2	21.9	11.9	27.5	19.3	28.6	22.7	9.9	36.2	8.8	20.2	52.7	8.5	85.9
	LH (IU/L)		6.7	8.7	9.7	13.9	20.1	16.3	28.4	6.5	3.1	24.3	3.9	10.0	31.2	4.9	25.1
	Hemoglobin (g/dL)		—	—	16.5	14.1	—	14.6	12.5	14.2	14	12.7	14.8	14.4	—	11.4	12.3
	Hematocrit (%)		—	—	49.2	41.7	—	44.5	39.2	42.7	41.2	39.6	45.3	43.6	—	35.1	36.6
Questionnaires	FACIT	Fatigue	9	11	16	9	7	10	23	30	7	8	8	10	23	25	15
	PHQ-9	Depression	1	1	9	0	2	1	5	13	3	1	1	0	10	9	2
	IIEF	Erectile Function	18	28	22	12	30	6	26	6	29	22	7	7	8	20	29
	DISF	Sexual Desire	19	31	20	29	21	25	18	9	20	14	9	18	11	28	21

**Figure 5:**
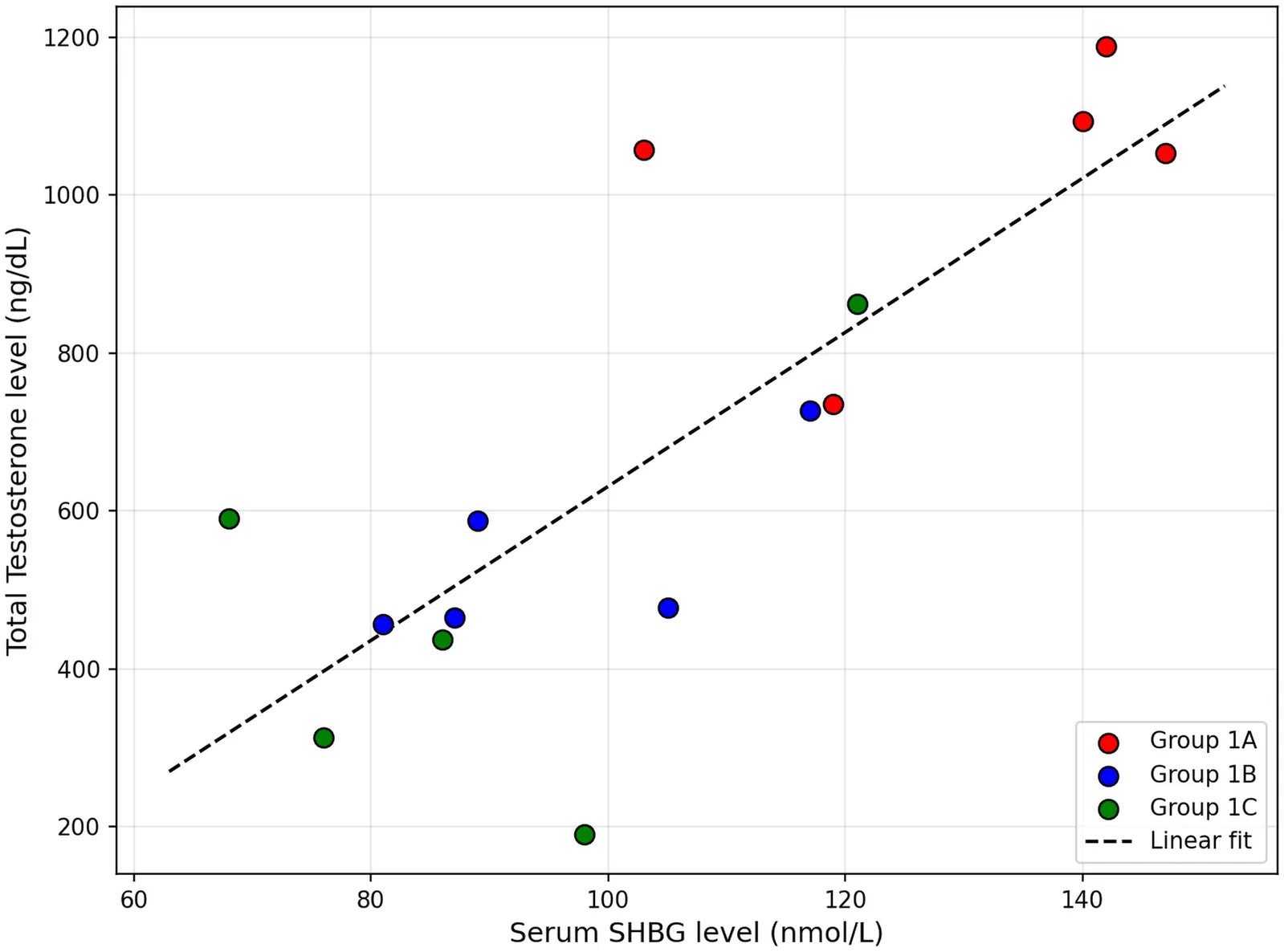
Association of SHBG with total testosterone concentrations in group 1.

An additional eight of 15 patients in group 1 had total testosterone level >450 ng/dL (but ≤916 ng/dL), a level that is substantially more than two SDs above the lower limit of the normal male range for healthy young men, who also met the criteria for primary testicular insufficiency listed above (group 1B) (1). The remaining three of 15 patients had total testosterone concentration that was low or low-normal (<450 ng/dL) (Group 1C, Fig. 2). Within Group 1, serum SHBG and total testosterone concentrations were strongly and positively associated (*R*^2^ = 0.622, *P* = .0005; Fig. 5).

Group 2 participants had high normal (n = 3) or above the reference range (n = 2) total testosterone concentration, low free testosterone, LH and FSH concentrations that were within the normal range for healthy young men, and one or more symptoms of hypogonadism. Their mean ± SD age was 59 ± 11 years; body mass 103.4 ± 25.1 kg; body mass index 35.7 ± 10.3 kg/m^2^; SHBG 119.0 ± 48.9 nmol/L; serum total testosterone 862.6 ± 354.1 ng/dL; free testosterone 38.2 ± 6.2 pg/mL; FSH 5.7 ± 0.7 U/L; LH 4.7 ± 1.5 U/L; estradiol 39.0 ± 24.3 pg/mL; and dihydrotestosterone 84.4 ± 40.9 ng/dL. The men in group 2 had higher body mass index (35.7 ± 10.3 kg/m^2^) than those in group 1 (24.9 ± 4.7 kg/m^2^). The two participants with total testosterone above the reference range had gynecomastia, normal testicular volume, moderate to severe fatigue and normal erectile function and libido. One had moderate depressive symptoms.

## Discussion

In this analysis, we describe the clinical and biochemical features of men with markedly elevated SHBG levels who exhibited unequivocally low free testosterone concentrations, measured using rigorously validated equilibrium dialysis assay. Despite unequivocally low free testosterone concentrations, some of these men showed mid- to high-normal or even total testosterone levels above the reference range, creating a striking divergence between total and free testosterone concentrations. Notably, all met predefined criteria for primary hypogonadism (1). These findings are consistent with free testosterone—rather than total testosterone—serving as the physiologically relevant regulator of the HPT feedback loop.

Circulating binding proteins are ubiquitous across plants and animals, playing indispensable roles in the transport, metabolism, and bioavailability of hormones, nutrients, and growth factors—a paradigm well-established in most biological disciplines, including endocrinology (2). Hemoglobin exemplifies this principle, binding oxygen in the lungs for targeted delivery and release in tissue capillaries, enabling unbound oxygen to diffuse into cells (17, 18). Yet, in andrology, the FHH—positing that unbound free testosterone is the biologically active fraction sensed by tissues—remains contentious despite broad acceptance elsewhere (9).

A primary critique of the FHH stems from epidemiologic studies and intervention trials where free testosterone correlates only modestly more strongly with androgen-dependent outcomes (eg, sexual function, muscle mass) than total testosterone, often failing statistical superiority (5, 7, 8, 19). As stated earlier, total and free testosterone exhibit high collinearity while both show merely modest associations with clinical endpoints. Our study circumvents this confound by examining men with markedly elevated SHBG and primary testicular dysfunction, in which total and free testosterone concentrations diverged dramatically. Here, unequivocally low free testosterone concentrations were associated with hypogonadal symptoms (eg, sexual symptoms, fatigue, depressive symptoms, and gynecomastia) and elevated LH/FSH—despite high normal or even testosterone concentrations above the reference range—consistent with FHH predictions. In an analysis of data from the European Male Aging Study, low free testosterone concentrations were associated with hypogonadal symptoms even in the presence of normal total testosterone concentrations (20).

The patients in Group 1A are paradigmatic: total testosterone concentrations in these men were above the reference range (>916 ng/dL), yet low free testosterone concentration in these men coincided with clinical hypogonadism features and elevated gonadotropins. Group 1B men, with total testosterone in the mid- to high-normal range (450-916 ng/dL, >2 SD above the lower limit per Travison et al (11)), similarly met the criteria for primary hypogonadism, supporting the importance of free testosterone over total testosterone in central biosensors. The elevated LH and/or FSH levels in patients in groups 1A and 1B are consistent with primary testicular dysfunction and with the HPT feedback loop sensing free testosterone levels rather than total testosterone to regulate LH and FSH secretion. Relying solely on total testosterone would have misclassified these men as eugonadal, potentially preventing consideration of testosterone replacement therapy.

The study has several strengths. The high SHBG levels reported in the electronic medical record were verified in our research laboratory using an extensively published assay. All hormone concentrations reported in the clinical record were confirmed by repeating the measurements in our research laboratory using rigorously validated assays in samples collected in the morning after an overnight fast. The rigorously–derived reference ranges of these assays have been published (10, 11); the free testosterone concentrations in all participants in group 1 were below the lower limit of free testosterone levels for healthy nonobese men as well as below the lower limit for healthy men 60 years or older (10); separate reference ranges for men >70 years are not available.

The proverbial “N-of-1” experiments of nature—rare phenotypes yielding mechanistic clarity—abound in medicine (eg, single-gene mutations revealing pathway functions). Though Groups 1A, 1B, and 1C were small (n = 15 combined), they are consistent with HPT feedback sensing free testosterone: LH/FSH remained unsuppressed despite elevated total testosterone, rising appropriately in relation to the low circulating free testosterone concentration despite high total testosterone, sensing the testosterone deficiency that may have also produced these men's symptoms and signs. The symptoms of testosterone deficiency, especially fatigue and depressive symptoms, though common in men with testosterone deficiency are also prevalent in middle-aged and older men with chronic diseases; however, in group 1, elevated LH and FSH concentrations together with these rigorously ascertained symptoms using validated questionnaires were consistent with primary testicular dysfunction in these men.

Several factors other than primary testicular dysfunction can influence gonadotropin secretion and merit consideration. In contrast to Group 1, the men in Group 2 had obesity (mean body mass index 35.7 kg/m^2^), and obesity can suppress hypothalamic–pituitary output and lower LH; their gonadotropin concentrations within the normal range despite low free testosterone may therefore reflect a functional, obesity-related suppression superimposed on reduced free testosterone rather than preserved testicular reserve (21). Estradiol, which is also largely SHBG-bound, exerts negative feedback on gonadotropin secretion in men; because we did not estimate free estradiol, a contribution of free estradiol to the observed LH and FSH concentrations cannot be ascertained (22).

More broadly, SHBG has been reported to modulate the gonadotropin response to sex-steroid feedback, consistent with a model in which the free, rather than the protein-bound, hormone fraction is sensed by central regulators (23-26). Notably, in transgenic mice that hyperexpressed the SHBG transgene and had markedly elevated circulating SHBG levels (23), serum LH and total testosterone levels were elevated and yet, the wet weights of androgen-sensitive tissues such as seminal vesicles ad *levator ani* muscle were lower than wild type mice. These mouse studies provide support to the notion that free but not total testosterone is associated with testosterone's action in androgen-sensitive tissues (23).

The differential diagnosis of markedly elevated SHBG with low free testosterone includes hepatic disease and cirrhosis, hyperthyroidism, use of anticonvulsants such as phenytoin, estrogen exposure, HIV infection, and rare variants in the SHBG gene (2). In older men, age-related increases in SHBG also contribute (27). Some of these conditions, such as cirrhosis, estrogen use, and HIV infection, may also be associated with hypogonadism. In the present cohort, identifiable contributors included cirrhosis, autoimmune hepatitis, metabolic dysfunction-associated steatotic liver disease, and phenytoin use, although no cause was identified in most participants. Recognizing this differential is clinically important, because in these conditions with elevated SHBG levels, sole reliance on total testosterone level could obscure the diagnosis.

Limitations include the study's small sample size due to the rarity of patients with markedly elevated SHBG levels and cross-sectional design limiting causality. Women were excluded due to distinct HPO-axis dynamics than that of the HPT-axis in men. We did not include men with elevated SHBG but normal free testosterone and normal LH/FSH, because in the absence of an orthogonal marker such as elevated LH and FSH, these individuals would not have provided a reliable comparator group for assessing testosterone deficiency or excess. In addition, although all Group 1 participants had elevated LH and/or FSH, serum gonadotropin concentrations also rise with age, and an age-related contribution to the elevated gonadotropins in our older participants cannot be fully excluded (28, 29). The small sample size precludes formal age-stratified analyses. These observations should be viewed as illustrative rather than representative of the broader population.

A key barrier to wider free testosterone adoption is the limited access to accurate equilibrium dialysis method, compounded by interlaboratory variability and variable nonharmonized reference ranges reported by commercial and academic laboratories. Although algorithms for estimating free testosterone concentrations from total testosterone, SHBG, and albumin concentrations have been published (30-33), such equations have inherent limitations because of the dynamic changes in the apparent binding affinity of SHBG and human serum albumin for sex hormones with varying sex hormone and binding protein concentrations (32, 34-36). The findings of our analyses provide a strong rationale for the generation of harmonized reference ranges for free testosterone concentration using standardized equilibrium dialysis procedure coupled with the measurements of testosterone in the dialysate using an LC-MS/MS assay certified by the HoST Program, such as that which we have previously published (10).

### Conclusions

In men with markedly elevated SHBG, low free testosterone concentrations were consistently associated with one or more symptoms and signs of testosterone deficiency and with elevated LH and/or FSH concentrations, even when total testosterone concentrations were within or above the reference range. Taken together, these data support the primacy of circulating free testosterone over total testosterone as the biologically relevant determinant of the systemic manifestations of testosterone deficiency, including the feedback regulation of gonadotropin secretion. Our findings provide a strong rationale for expanding access to accurate, validated free testosterone assays with rigorously derived, harmonized reference ranges to improve diagnostic precision and patient care.

## Data Availability

Original data generated and analyzed during the study are included in the published article.

## References

[1] Bhasin S, Brito JP, Cunningham GR, et al Testosterone therapy in men with hypogonadism: an endocrine society clinical practice guideline. J Clin Endocrinol Metab. 2018;103(5):1715‐1744. 29562364 10.1210/jc.2018-00229

[2] Goldman AL, Bhasin S, Wu FCW, Krishna M, Matsumoto AM, Jasuja R. A reappraisal of testosterone's binding in circulation: physiological and clinical implications. Endocr Rev. 2017;38(4):302‐324. 28673039 10.1210/er.2017-00025 PMC6287254

[3] Hammond GL . Access of reproductive steroids to target tissues. Obstet Gynecol Clin North Am. 2002;29(3):411‐423. 12353665 10.1016/s0889-8545(02)00008-6

[4] Mendel CM . The free hormone hypothesis: a physiologically based mathematical model. Endocr Rev. 1989;10(3):232‐274. 2673754 10.1210/edrv-10-3-232

[5] Corona G, Isidori AM, Buvat J, et al Testosterone supplementation and sexual function: a meta-analysis study. J Sex Med. 2014;11(6):1577‐1592. 24697970 10.1111/jsm.12536

[6] Snyder PJ, Bhasin S, Cunningham GR, et al Effects of testosterone treatment in older men. N Engl J Med. 2016;374(7):611‐624. 26886521 10.1056/NEJMoa1506119 PMC5209754

[7] Cunningham GR, Stephens-Shields AJ, Rosen RC, et al Testosterone treatment and sexual function in older men with low testosterone levels. J Clin Endocrinol Metab. 2016;101(8):3096‐3104. 27355400 10.1210/jc.2016-1645 PMC4971331

[8] Pencina KM, Travison TG, Cunningham GR, et al Effect of testosterone replacement therapy on sexual function and hypogonadal symptoms in men with hypogonadism. J Clin Endocrinol Metab. 2024;109(2):569‐580. 37589949 10.1210/clinem/dgad484

[9] Anawalt BD, Handelsman DJ. Debate about the free testosterone hypothesis: useful tool or misguided mirage? J Clin Endocrinol Metab. 2025;110(10):e3536‐e3540. 40581344 10.1210/clinem/dgaf370

[10] Jasuja R, Pencina KM, Spencer DJ, et al Reference intervals for free testosterone in adult men measured using a standardized equilibrium dialysis procedure. Andrology. 2023;11(1):125‐133. 36251328 10.1111/andr.13310

[11] Travison TG, Vesper HW, Orwoll E, et al Harmonized reference ranges for circulating testosterone levels in men of four cohort studies in the United States and Europe. J Clin Endocrinol Metab. 2017;102(4):1161‐1173. 28324103 10.1210/jc.2016-2935 PMC5460736

[12] Huang G, Bhasin S, Pencina K, Cheng M, Jasuja R. Circulating dihydrotestosterone, testosterone, and free testosterone levels and dihydrotestosterone-to-testosterone ratios in healthy women across the menstrual cycle. Fertil Steril. 2022;118(6):1150‐1158. 36371319 10.1016/j.fertnstert.2022.09.011

[13] Rosen RC, Cappelleri JC, Gendrano N 3rd. The International Index of Erectile Function (IIEF): a state-of-the-science review. Int J Impot Res. 2002;14(4):226‐244. 12152111 10.1038/sj.ijir.3900857

[14] Derogatis LR . The Derogatis Interview for Sexual Functioning (DISF/DISF-SR): an introductory report. J Sex Marital Ther. 1997;23(4):291‐304. 9427208 10.1080/00926239708403933

[15] Cella D, Eton DT, Lai JS, Peterman AH, Merkel DE. Combining anchor and distribution-based methods to derive minimal clinically important differences on the Functional Assessment of Cancer Therapy (FACT) anemia and fatigue scales. J Pain Symptom Manage. 2002;24(6):547‐561. 12551804 10.1016/s0885-3924(02)00529-8

[16] Kroenke K, Spitzer RL, Williams JB. The PHQ-9: validity of a brief depression severity measure. J Gen Intern Med. 2001;16(9):606‐613. 11556941 10.1046/j.1525-1497.2001.016009606.x PMC1495268

[17] Antonini E, Brunori M. Hemoglobin and Myoglobin in Their Reactions with Ligands. North-Holland Pub. Co; 1971.

[18] Olson JS . Kinetic mechanisms for O(2) binding to myoglobins and hemoglobins. Mol Aspects Med. 2022;84:101024. 34544605 10.1016/j.mam.2021.101024 PMC8821315

[19] Bhasin S, Snyder PJ. Testosterone treatment in middle-aged and older men with hypogonadism. N Engl J Med. 2025;393(6):581‐591. 40768717 10.1056/NEJMra2404637

[20] Antonio L, Wu FC, O'Neill TW, et al Low free testosterone is associated with hypogonadal signs and symptoms in men with normal total testosterone. J Clin Endocrinol Metab. 2016;101(7):2647‐2657. 26909800 10.1210/jc.2015-4106

[21] Muir CA, Wittert GA, Handelsman DJ. Approach to the patient: low testosterone concentrations in men with obesity. J Clin Endocrinol Metab. 2025;110(9):e3125‐e3130. 40052430 10.1210/clinem/dgaf137 PMC12342380

[22] Russell N, Grossmann M. MECHANISMS IN ENDOCRINOLOGY: estradiol as a male hormone. Eur J Endocrinol. 2019;181(1):R23‐R43. 31096185 10.1530/EJE-18-1000

[23] Laurent MR, Hammond GL, Blokland M, et al Sex hormone-binding globulin regulation of androgen bioactivity in vivo: validation of the free hormone hypothesis. Sci Rep. 2016;6(1):35539. 27748448 10.1038/srep35539 PMC5066276

[24] de Ronde W, van der Schouw YT, Muller M, et al Associations of sex-hormone-binding globulin (SHBG) with non-SHBG-bound levels of testosterone and estradiol in independently living men. J Clin Endocrinol Metab. 2005;90(1):157‐162. 15509641 10.1210/jc.2004-0422

[25] Winters SJ . SHBG and total testosterone levels in men with adult onset hypogonadism: what are we overlooking? Clin Diabetes Endocrinol. 2020;6(1):17. 33014416 10.1186/s40842-020-00106-3 PMC7526370

[26] de Ronde W, van der Schouw YT, Pierik FH, et al Serum levels of sex hormone-binding globulin (SHBG) are not associated with lower levels of non-SHBG-bound testosterone in male newborns and healthy adult men. Clin Endocrinol (Oxf). 2005;62(4):498‐503. 15807883 10.1111/j.1365-2265.2005.02252.x

[27] Liu PY, Beilin J, Meier C, et al Age-related changes in serum testosterone and sex hormone binding globulin in Australian men: longitudinal analyses of two geographically separate regional cohorts. J Clin Endocrinol Metab. 2007;92(9):3599‐3603. 17595245 10.1210/jc.2007-0862

[28] Wu FC, Tajar A, Pye SR, et al Hypothalamic-pituitary-testicular axis disruptions in older men are differentially linked to age and modifiable risk factors: the European Male Aging Study. J Clin Endocrinol Metab. 2008;93(7):2737‐2745. 18270261 10.1210/jc.2007-1972

[29] Xia F, Wang N, Han B, et al Hypothalamic-pituitary-gonadal axis in aging men and women: increasing total testosterone in aging men. Neuroendocrinology. 2017;104(3):291‐301. 27178254 10.1159/000446656

[30] Vermeulen A, Verdonck L, Kaufman JM. A critical evaluation of simple methods for the estimation of free testosterone in serum. J Clin Endocrinol Metab. 1999;84(10):3666‐3672. 10523012 10.1210/jcem.84.10.6079

[31] Södergård R, Bäckström T, Shanbhag V, Carstensen H. Calculation of free and bound fractions of testosterone and estradiol-17 beta to human plasma proteins at body temperature. J Steroid Biochem. 1982;16(6):801‐810. 7202083 10.1016/0022-4731(82)90038-3

[32] Zakharov MN, Bhasin S, Travison TG, et al A multi-step, dynamic allosteric model of testosterone's binding to sex hormone binding globulin. Mol Cell Endocrinol. 2015;399:190‐200. 25240469 10.1016/j.mce.2014.09.001

[33] Ly LP, Handelsman DJ. Empirical estimation of free testosterone from testosterone and sex hormone-binding globulin immunoassays. Eur J Endocrinol. 2005;152(3):471‐478. 15757865 10.1530/eje.1.01844

[34] Jasuja R, Spencer D, Jayaraj A, et al Estradiol induces allosteric coupling and partitioning of sex-hormone-binding globulin monomers among conformational states. iScience. 2021;24(6):102414. 34041454 10.1016/j.isci.2021.102414 PMC8144348

[35] Jayaraj A, Schwanz HA, Spencer DJ, et al Allosterically coupled multisite binding of testosterone to human serum albumin. Endocrinology. 2021;162(2):bqaa199. 33125473 10.1210/endocr/bqaa199 PMC7774055

[36] Anees M, Fugate MK, Bhasin S, Pandey D, Chowdhury S, Jasuja R. Multiple dynamically-coupled binding sites on human serum albumin regulate estradiol's nonlinear binding. Endocrinology. 2026;167(4):bqag027. 41817210 10.1210/endocr/bqag027

